# Case report of long-term survival with metastatic triple-negative breast carcinoma: Treatment possibilities for metastatic disease

**DOI:** 10.1097/MD.0000000000015302

**Published:** 2019-04-19

**Authors:** Ben Man-Fai Chue, Bryce Douglas La Course

**Affiliations:** Lifespring Cancer Treatment Center, Seattle, WA.

**Keywords:** immunotherapy, long-term survival, metastatic triple-negative breast cancer, metronomic chemotherapy, sequential chemotherapy regimens

## Abstract

**Rationale:**

Breast cancer is the most common as well as one of the most devastating cancers among women in the United States. Prognosis is poor for patients with metastatic breast cancer, especially for patients with so-called “triple-negative” disease. The lack of effective therapies for metastatic triple-negative breast cancer outlines the need for novel and innovative treatment strategies.

**Patient concerns:**

A 58-year-old underwent a mastectomy which revealed a recurrent triple-negative breast carcinoma. Afterward, she presented with a growing mass in her left axilla and chest wall. A computed tomography scan showed axillary and supraclavicular adenopathy, nodules in the left upper and lower lobe of the lungs, and 2 areas of disease in the liver. A bone scan showed lesions in the ribs.

**Diagnosis:**

The patient was diagnosed with a recurrent metastatic triple-negative breast carcinoma that spread to the lung, liver, and bones.

**Interventions:**

The patient was treated with metronomic chemotherapy, sequential chemotherapy regimens, and immunotherapy.

**Outcomes:**

The patient is now over 15 years out from her diagnosis of metastatic disease without any evidence of recurrent disease, likely due to the patient's treatment strategy which included sequential metronomic chemotherapy regimens and immunotherapy.

**Lessons:**

Sequential metronomic chemotherapy regimens in combination with immunotherapy might be an effective treatment option for patients with metastatic triple-negative breast cancer. We hope that this case can provide some guidance for the treatment of metastatic triple-negative breast cancer and motivate research that can potentially lead to more cases of long-term survival for patients who develop this dismal disease.

## 1 Introduction

Breast cancer remains the most common cancer diagnosed among women in the United States and is the second leading cause of cancer-related deaths, with approximately 41,000 patients projected to die from this disease in 2018 alone.^[1]^ The prognosis for patients with metastatic breast cancer varies based on many factors including estrogen receptor (ER), progesterone receptor (PR), and human epidermal growth factor receptor 2 (HER2) status. Tumors that do not express the ER, PR, or HER2 receptors are known as “triple-negative” breast cancers (TNBCs) and represent approximately 11% of all breast cancers.^[2]^ This subtype of breast cancer is known for being aggressive, having a high probability of distant recurrence after adjuvant therapy, and progressing quickly on palliative chemotherapy treatment in the metastatic setting.^[2,3]^ Patients with metastatic TNBC have a poor prognosis, with a median overall survival of 13.3 months with treatment.^[3]^ Continuing chemotherapy treatment until disease progression is currently the standard of care for patients with metastatic TNBC, with no preferred chemotherapy regimens established at this time. The lack of effective therapies for this aggressive disease highlights the need for the development of novel treatment strategies. Here we report the case of a patient with metastatic TNBC that metastasized to the lungs, liver, and bones who achieved long-term remission without evidence of disease recurrence after 15 years. Her case is followed by a discussion of the treatment strategy which likely has led to her remarkable survival.

## 2 Case report

A Caucasian female initially presented with a nodule in her left breast in March 2001 at the age of 56. Before this, the patient was a homemaker with a relatively unremarkable past medical history aside from some mild arthritis. Her father and brother had prostate cancer, and her mother apparently had uterine cancer. Her social history was negative for tobacco, alcohol as well as illicit drug use. A core biopsy performed in mid-April 2001 showed a high-grade infiltrating ductal carcinoma, with a Bloom–Richardson score of 9/9. Immunohistochemistry showed that the tumor was positive for ER expression and negative for PR expression and HER2 overexpression. The patient underwent a left lumpectomy with an axillary lymph node dissection shortly after the biopsy. Pathology revealed a 2.0 × 1.8 × 1.3 cm high-grade invasive ductal carcinoma. 0 of the 15 left axillary lymph nodes examined were positive for carcinoma, and no lymphatic invasion was identified. The patient declined adjuvant chemotherapy and radiation therapy.

**Figure 1:**
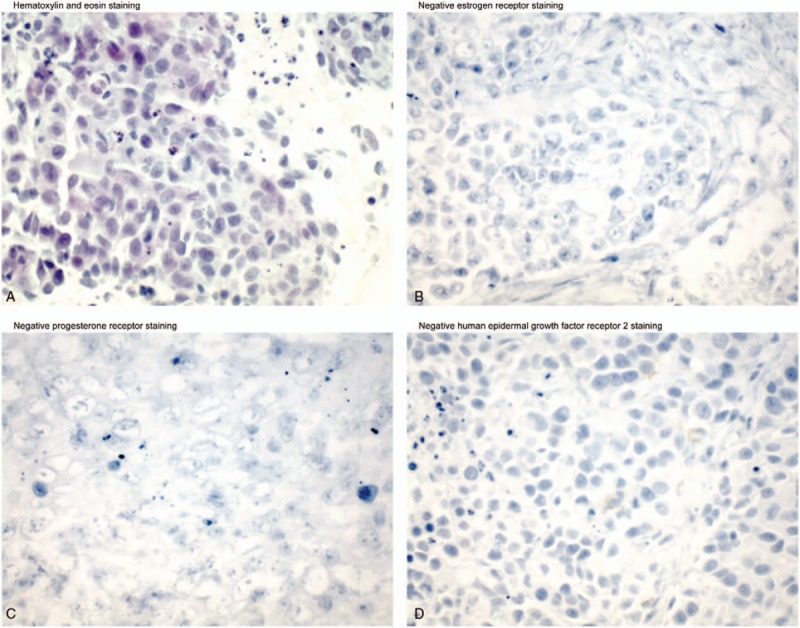
An analysis of the original biopsy specimen of the left breast mass from May 2003 revealed a poorly differentiated carcinoma seen with hematoxylin and eosin staining (A). Immunohistochemical staining of the biopsy sections showed that the tumor was negative for estrogen receptor expression (B) negative for progesterone receptor expression (C), and negative for human epidermal growth factor receptor 2 overexpression (D).

In the spring of 2003, the patient noticed another mass under the nipple of her left breast. An ultrasound-guided left breast biopsy was performed in early May 2003 which revealed a high-grade ductal carcinoma. The patient then underwent bilateral simple mastectomies to remove the left breast tumor in late May 2003. Pathology of the left breast mass showed a poorly-differentiated carcinoma that measured 3.0 × 3.5 × 2.0 cm with a Bloom–Richardson score of 9/9. The tumor was negative for ER, PR, HER2 by immunohistochemistry, and for amplification of the *HER2* gene by fluorescence in situ hybridization, indicating she had triple-negative recurrent disease (Fig. 1). The patient again declined any treatment.

In early September 2003, the patient noticed a growing mass in her left axilla and chest wall. A computed tomography (CT) scan of the chest showed a 3.8 cm necrotic left axillary lymph node with axillary and supraclavicular adenopathy. Nodules were also seen in the left upper and lower lobe of the lungs along with 2 subtle areas of low attenuation in the liver. A bone scan performed in December 2003 revealed bone lesions along the left 3rd and 6th ribs posterolaterally. The patient was concluded to have metastatic disease to the lungs, liver, and bones. Unfortunately, these images have been destroyed by the imaging facility and cannot be used in this publication. The details of the scans were obtained from the official written scan reports.

After consulting with the patient, she decided to proceed with chemotherapy treatment. She was given 4 sequential chemotherapy regimens (Table 1) to try and control her cancer along with monthly zoledronic acid to try to prevent bone complications due to her osseous metastases. Granulocyte-macrophage-colony-stimulating factor (GM-CSF) was used throughout the treatment to prevent or treat chemotherapy-induced neutropenia as well as stimulate the immune system. After 14 doses of weekly paclitaxel and carboplatin, there was remarkable tumor shrinkage in the left chest wall and axillary area on her physical examinations. Her cancer antigen (CA) 27.29 (normal range: <38 U/mL) also decreased from a pretreatment level of 52.1 to 19.8 in mid-April 2004 which was consistent with the patient's physical exam findings. The patient's CA 27.29 then remained within normal range from this point onward. The patient was then switched to weekly doxorubicin liposome in June 2004. Twelve doses were planned, but she only received 8 doses due to developing palmar-plantar erythrodysesthesia. Her symptoms resolved after the drug was withdrawn in late July 2004. The patient then continued chemotherapy treatment and received 6 doses of weekly gemcitabine and cisplatin from August 2004 to October 2004. The patient developed thrombocytopenia and her second to the last dose of this regimen was given with a reduced dose of cisplatin. Gemcitabine was dose reduced during her last infusion of this regimen. She was then switched to weekly vinorelbine in early December 2004 and completed 12 doses of chemotherapy treatment in late February 2005.

**Table 1:**
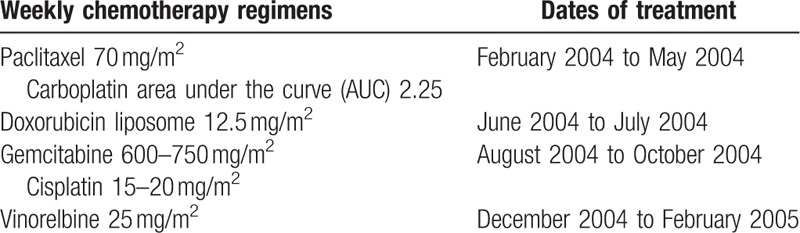
Treatment history and dosing.

Aside from developing neutropenia and thrombocytopenia secondary to chemotherapy treatment and palmar-plantar erythrodysesthesia secondary to doxorubicin liposome, the patient tolerated her treatment relatively well. A CT scan performed in March 2005 showed no evidence of disease in the lung and liver. The patient was taken off of chemotherapy treatment but continued on monthly zoledronic acid and pursued a watchful waiting approach from February 2005 to June 2005.

In June 2005 the patient developed several small skin nodules in her left axillary chest wall. A positron emission tomography (PET) and CT scan performed shortly after showed minimal thickening of the left chest wall, suspicious for recurrent disease, although no biopsy was performed. There was no other evidence of metastatic disease. She was started on imiquimod cream, which was applied on the skin nodules, for immune stimulation. The patient reported pruritus and erythema in the applied area, but she did not complain of having any pain. These nodules resolved after a few weeks. In October 2005, the patient noticed several pea-sized nodules in her left axilla which were also suspicious for disease recurrence. The patient continued applying imiquimod cream to the area. She developed erythema, ulceration, and skin breakdown in that area, but this resolved after stopping imiquimod. The skin nodules resolved by January 2006. The patient's condition continued to improve, and the frequency of her zoledronic acid infusions was reduced to every other month in May 2006, and then quarterly in September 2007. In December 2008 she continued with biannual infusions of zoledronic acid. A germline BRCA1 and BRCA2 analysis performed in December 2009 did not detect any mutations. Routine PET/CT and CT scans continue to show no evidence of recurrent or persistent metastatic disease. The patient is now 73 years old and is enjoying a good quality of life. She is currently over 15 years out from her diagnosis of recurrent metastatic TNBC.

## 3 Discussion

The prognosis of this patient before starting treatment was particularly poor, not only because her tumor did not express the ER, PR, or HER2 receptors, but also because she had stage IV disease with multiple visceral metastases. With the typical prognosis of metastatic TNBC being slightly over 1 year, the 15-year survival of this patient is quite remarkable, especially given that she is currently free of disease and has not received any chemotherapy treatment since late February 2005. To our knowledge, this patient is the longest reported survivor of metastatic TNBC. Her long-term survival without recurrence suggests that this patient may be cured of a cancer that is not thought to be curable. We believe that our treatment methodology, which included using metronomic chemotherapy, switching chemotherapy regimens before anticipated disease progression, and utilizing immune therapies, all contributed to her outstanding survival. Below, we will describe each of these treatment strategies in detail.

### 3.1 Metronomic chemotherapy

The dosing of a standard chemotherapy regimen is based on a maximum tolerated dose (MTD). This dose is typically the highest possible dose that is not lethal to the patient. The idea of an MTD was originally developed with the logic that “more is better” to try and maximize the amount of cancer cell death. A high dose of chemotherapy can kill cancer cells, but due to the relatively nonspecific mechanism of action of most chemotherapy agents, this high dose of chemotherapy can also result in clinical toxicities which is why standard chemotherapy regimens are often administered every 3 weeks. The breaks in between standard chemotherapy doses are crucial for the recovery of normal tissues, but logically this can also give time for cancer cells to grow and progress as well. Thus, the dosing of chemotherapy agents and the frequency of chemotherapy administration may play an important role in the efficacy of treatment as well as the patient's quality of life.

This patient received lower doses of chemotherapy on a more frequent basis, also known as “metronomic chemotherapy.” Although lower doses of chemotherapy agents were given to this patient during each administration, the overall dose intensity (the total dose of chemotherapy administered per unit time) of her chemotherapy agents was a similar, if not higher, dose intensity, compared to the standard dosing of each respective chemotherapy agent. Studies have shown that reducing dose intensity, most commonly due to myelosuppression, correlates with poorer disease-free survival and overall survival, while maintaining a relatively high planned dose intensity is associated with better clinical outcomes.^[4]^ Due to the lower doses used during each administration, metronomic chemotherapy regimens can minimize severe adverse events and prolonged drug-free breaks. In addition, a more steady dosing schedule may actually kill more cancer cells by maintaining a more constant drug concentration in the body. This logic may explain why dose-dense regimens (chemotherapy regimens with an increased frequency of administration) have been shown to be more effective in the treatment of several kinds of cancer, including breast cancer, when compared to standard treatment.^[5]^ We have also treated pancreatic cancer patients using a similar dosing strategy, which has yielded exciting results.^[6,7]^

The main mechanism of action of metronomic chemotherapy was initially thought to be its effects on endothelial cells resulting in antiangiogenic effects. There is a fair amount of literature that suggests that paclitaxel has antiangiogenic effects when administered in lower doses more frequently.^[8]^ Likely due to their aggressive nature, TNBCs are known to have enhanced angiogenesis.^[9]^ The proangiogenic tumor microenvironment creates an abnormal vascular network that can result in increased interstitial pressure and decrease drug penetration, ultimately decreasing the efficacy of systemic treatment.^[10]^ By blocking angiogenesis and normalizing the tumor vasculature, more chemotherapy can reach the tumor, potentially improving the efficacy of treatment.

In addition to having direct cytotoxic and antiangiogenic effects, it has been discovered that metronomic chemotherapy can also have antistromal and immunostimulatory effects.^[11–13]^ There are even some thoughts that the effects that metronomic chemotherapy has on the tumor microenvironment can decrease the rate of acquired chemotherapy resistance.^[11,12]^ Targeting both cancer cells and the tumor microenvironment may play an important role in the future of cancer treatment.

### 3.2 Sequential chemotherapy regimens

The standard way to administer chemotherapy treatment is to continue a single chemotherapy regimen until noticeable disease progression. Patients with metastatic TNBC tend to relapse quickly on chemotherapy treatment, likely due to acquired disease resistance.^[3]^ Drug resistance is seen as the primary cause of failure of chemotherapy treatment for cancer and continuing a chemotherapy regimen until disease progression will inevitably breed chemotherapy-resistant disease. Switching chemotherapy regimens before disease progression, as we did for this patient, may prevent the development of disease resistance, allowing for a continual decrease in the number of cancer cells, and perhaps a better chance of achieving long-term survival.

Tumors are known to have a large amount of genetic diversity, even within a single mass, and chemotherapy treatment can induce strong selective pressure for cells that have intrinsic or acquired mutations which can resist treatment.^[14]^ Switching chemotherapy agents may eradicate cancer cells that developed resistance to the cytotoxic agents in the previous regimen, especially if the new chemotherapy agents have a different mechanism of action. There are even some suggestions that cancer cells can become dependent on certain therapies after long-term drug exposure, and switching the drugs used may increase treatment efficacy by inducing cell death of the cancer cells that have become “addicted” to the previous therapy.^[12,13]^ The idea of switching chemotherapy treatments before the development of disease resistance has broad implications and could transform the idea of cancer being an acute disease to more of a chronic illness. In addition to potentially increasing treatment efficacy and preventing the development of disease resistance, switching treatment regimens can also help prevent the accumulation of chemotoxicity from a single chemotherapy regimen, which can improve the quality of life of patients receiving treatment.

This idea of sequential chemotherapy regimens has been successfully introduced in the treatment of metastatic non-small cell lung carcinoma. Patients who responded to first-line chemotherapy and pursued a switch maintenance therapy were found to have improved overall survival compared to placebo or observation, and switch maintenance therapy was also less toxic compared to continuous maintenance therapy.^[15]^ A similar treatment strategy has also been applied and has found major success in the treatment of pediatric acute lymphoblastic leukemia (ALL). A diagnosis of ALL was fatal for children in the 1950s. Currently, this disease has a cure rate of more than 80% in children. The current treatment for ALL involves several combination chemotherapy regimens that are given sequentially to eliminate any remaining disease.^[16]^ Sequential chemotherapy regimens in ALL has been considered one of the greatest achievements in the field of oncology to date.

This patient received several different chemotherapy regimens sequentially (Table 1). Her first regimen consisted of paclitaxel and carboplatin. Paclitaxel that is administered on a weekly basis has been shown to be superior to paclitaxel that is administered every 3 weeks in the treatment of metastatic breast cancer, with increased response rates, time to progression, as well as survival.^[17]^ Moreover, there is some evidence that platinum-based chemotherapy regimens improve the overall survival of patients with metastatic TNBC.^[18]^ The addition of platinum agents to treatment regimens has likely been slow to catch on due to the significant toxicity of the standard doses of these agents. However, the carboplatin dose of AUC 2.25 and cisplatin dose of 15 to 20 mg/m^2^ that was given on a weekly basis were tolerated relatively well by this patient.

After she received 14 doses of paclitaxel and carboplatin, she was switched to weekly doxorubicin liposome. Anthracyclines are known to be very effective in the treatment of breast cancer, but carry a risk of cardiotoxicity and significant myelosuppression. We prefer to use doxorubicin liposome instead of doxorubicin because doxorubicin liposome has a favorable toxicity profile, less hematological toxicity, and has been found to cause less cardiotoxicity compared to doxorubicin.^[19]^ This is an important consideration to reduce potential comorbidities in the future, especially if patients have the potential to achieve long-term survival. The patient tolerated treatment with weekly doxorubicin liposome well aside from developing palmar-plantar erythrodysesthesia, but perhaps this treatment might be more tolerable if it was administered every 2 weeks due to doxorubicin liposome's relatively long half-life. This treatment might also be more effective if it was combined with weekly paclitaxel.

The patient's next regimen, gemcitabine, and cisplatin, has been shown to improve outcomes in patients with metastatic TNBC compared to patients without metastatic TNBC.^[20]^ The patient also tolerated this regimen well aside from developing thrombocytopenia, but maybe this could be lessened by starting with a dose of gemcitabine 600 mg/m^2^ and cisplatin 15 mg/m^2^ instead of gemcitabine 750 mg/m^2^ and cisplatin 20 mg/m^2^, which is what we have done subsequently with other patients. Her final regimen consisted of vinorelbine, which is another effective drug for the treatment of metastatic breast cancer patients who have been exposed to anthracyclines and taxanes in previous treatments.^[21]^ Since the patient's diagnosis 15 years ago, there are several new treatments available that may be more effective than vinorelbine, such as eribulin or irinotecan.

By giving this series of effective treatments sequentially, we believe that this prevented disease resistance and allowed the patient to achieve complete remission after approximately 1 year of treatment. The combination of agents was chosen to avoid overly additive side effects, such as myelosuppression, so that the patient could receive continuous treatment without interruption and also maintain a relatively high overall dose intensity. In regards to the order of these regimens, it is unknown whether or not this is the most optimum sequence of regimens and this should be further investigated.

### 3.3 Immunotherapy

More evidence is accumulating suggesting that some subtypes of metastatic TNBC can be particularly responsive to immunotherapy, with some studies showing promising results.^[22]^ However, cancers can escape an antitumor immune response in several ways such through the upregulation of regulatory T cells (Tregs) and secretion of immunosuppressive cytokines into the tumor microenvironment as well as through the expression of immunosuppressive proteins, such as programmed death-ligand 1, on the cell surface.^[22–24]^ The situation is further exacerbated by the immunosuppressive effect of standard dose chemotherapy.^[25]^ When the immunosuppressive activity of the tumor outweighs the body's antitumor immune response, this is thought to promote tumor progression.

Metronomic chemotherapy, in addition to having a lesser impact on blood counts, is thought to have immunomodulatory properties. In preclinical studies, low-dose paclitaxel and gemcitabine have been shown to decrease the number and viability of Tregs as well as myeloid-derived suppressor cells in the tumor microenvironment, which could potentially allow for a more potent antitumor immune response.^[26]^ Moreover, the antiangiogenic effects of metronomic chemotherapy may be synergistic with its immunostimulatory properties. Normalizing the tumor vasculature could allow the immune system to better reach the tumor bed, just as it is thought to allow more chemotherapy treatment to reach the tumor. Interestingly, the patient also received zoledronic acid due to her bone metastases, which may also have immunomodulatory properties. This effect seems to be more prevalent in ER-positive breast cancer cells, but more research will need to be conducted to assess the immunomodulatory properties in ER-negative breast cancer.^[27]^

The patient also received several immunostimulatory agents which may have played a role in her long disease-free remission. While receiving chemotherapy treatment, the patient received GM-CSF to prevent or treat chemotherapy-induced neutropenia. We prefer the use of GM-CSF compared to other colony-stimulating factors because GM-CSF stimulates both the granulocyte as well as the monocyte/macrophage and dendritic cell (DC) lines, while other colony-stimulating factors only stimulate the granulocyte cell line. DCs are crucial for antigen presentation and activation of the adaptive immune system and macrophages can remove dead tumor cells via phagocytosis. Moreover, preclinical evidence suggests that low-dose paclitaxel can stimulate DC maturation and the anthracyclines can promote the phagocytosis of tumor cells by DCs, suggesting a synergistic role of GM-CSF in combination with 2 of the patient's chemotherapy regimens.^[28]^

The patient also applied imiquimod topically to small superficial lesions in her left axillary region that were likely secondary to recurrent disease. Imiquimod has known antitumor activity in superficial basal cell carcinoma and it is thought to work by stimulating the innate and adaptive immune system in the applied area via toll-like receptor 7.^[29]^ The patient's left axillary lesions resolved after she applied imiquimod cream to the area, suggesting that imiquimod may be able to be used to treat superficial metastases from TNBC. One small study also had similar findings, and it would be interesting to investigate whether metastatic TNBC was more responsive to local immunotherapy than other breast cancer types.^[30]^ Perhaps the success of imiquimod in this patient was due to the minimal tumor burden the patient had at the time, especially considering that a smaller tumor size in basal cell carcinoma is correlated with a more favorable prognosis after treatment with imiquimod.

## 4 Conclusion

The patient described in this case report is now 15 years out from her diagnosis of recurrent metastatic TNBC without evidence of persistent or recurrent metastatic disease. Her treatment, which included the use of sequential metronomic chemotherapy regimens as well as several immunotherapies, was tolerated relatively well and likely contributed to her remarkable survival. Although this is only 1 case, we have treated another patient with metastatic TNBC with a similar strategy who is now over 6 years out and free of disease as well as a few other patients who achieved longer than average survivals. We are planning to publish this data in a small case series in the future. We hope that this encouraging case can offer hope to those who are suffering from this debilitating disease and spark the formation of larger clinical trials to further evaluate this treatment strategy due to the potential significant medical, psychological, and economic implications. These ideas not only have the possibility to shift the paradigm of treating metastatic TNBC, but also potentially other metastatic cancers as well.

## Acknowledgments

We would like to thank Max Tse and Jeffrey Wang for drafting earlier versions of this paper, and Emmanuel De Dios, without whom none of this work could have been done.

## Author contributions

**Conceptualization:** Ben Man-Fai Chue, Bryce Douglas La Course.

**Formal analysis:** Ben Man-Fai Chue, Bryce Douglas La Course.

**Writing – original draft:** Ben Man-Fai Chue, Bryce Douglas La Course.

**Writing – review and editing:** Ben Man-Fai Chue, Bryce Douglas La Course.
